# Native low density lipoprotein induces pancreatic β cell apoptosis through generating excess reactive oxygen species

**DOI:** 10.1186/1476-511X-10-123

**Published:** 2011-07-26

**Authors:** Xiuli Lu, Jianli Liu, Xiangyu Cao, Xiao Hou, Xude Wang, Chenguang Zhao, Youliang Wang, Yang Li, Hisao Seo, Bing Gao

**Affiliations:** 1 School of Life Science, Liaoning University, Shenyang 110036, China; 2 College of Life and Health Science, Chubu University, Aichi, 487-8501, Japan; 3 Institute of Basic Medical Sciences, Shenyang Medical College, Shenyang, 110034, China

**Keywords:** LDLS, MIN6, apoptosis, ROS

## Abstract

**Background:**

The growing evidences demonstrated hyperlipidemia in obesity and type 2 diabetes is characterized by high levels of free fatty acids, low-density lipoprotein (LDL), triglyceride, and cholesterol.

**Method and Results:**

We investigated the effect of LDL particles (LDLs) loading on MIN6 cells derived from pancreatic β cells. Exposure of MIN6 cells to LDLs induced apoptosis in dose and time-dependent manner, demonstrated by the TUNEL in situ apoptotic assay. The immunocytochemical analysis and Western blotting revealed that the LDLs-induced apoptosis is associated with the activation of caspase 3 and upregulation of p53. The intracellular concentration of Reactive Oxygen Species (ROS) measured by use of DCFHDA was significantly increased after loading LDLs, demonstrating the induced apoptosis by LDLs loading was mediated through oxidative stress. Addition of reduced form of Glutathione (GSH) in the medium rescued MIN6 cells from apoptosis. The Cellular cholesterol level was increased significantly after LDLs loading, suggesting that the excess cholesterol induced by LDLs loading might contribute to the apoptosis in MIN6s. Agarose electrophoresis demonstrated that the LDLs added to the medium were not oxidized.

**Conclusion:**

Taken together, these results demonstrate that the LDLs loading can induce apoptosis of MIN6 cells mediated by the excess cellular cholesterol and generation of oxidative stress.

## Background

The growing evidences showing hyperlipidemia in obesity and type 2 diabetes (T2D) is characterized by high levels of free fatty acids, low-density lipoprotein (LDL), triglyceride, and cholesterol [1]. It is of note that rat and human β-cells express high-affinity LDL receptors leading to lipid accumulation in β-cells [2,3]. It was also reported LDL causes rat β-cell death [2]. However, how LDL exerts toxic effect on β-cells is still not fully understood.

Our previous study has demonstrated that the free cholesterol loading induces apoptosis of MIN6 pancreatic β cells (MIN6s) through the generation of excess of reactive oxygen species (ROS) and activation of p38/JNK MAP kinase-stress signaling pathway [4]. To confirm the physiological significance of the cholesterol cytotoxicity in pancreatic β cells, we studied the effect of LDLs loading in MIN6s.

## Methods

### Cell line

MIN6 cells were kindly donated from Prof. J. -I. Miyazaki (University of Osaka, Osaka, Japan). MIN6 cells were grown in Dulbecco's modified Eagle's medium (DMEM, 25 mmol/l glucose) equilibrated with 5% CO_2 _and 95% air at 37 C. The medium was supplemented with 10% fetal bovine serum, 50 mg/l streptomycin and 75 mg/1 penicillin sulphate. MIN6 cells used in the present study were harvested at passages 16-23. When indicated the cells were incubated with diluted CLC at different time intervals. The sterol contents in the whole cell extracts were determined by an enzymatic cholesterol assay kit. When MIN6 cells were incubated with LDLs and 2 mM reduced glutathione (LDLs/GSH), they were incubated for 48 hours. The Human Low-Density Lipoproteins (LDLs) were purchased from ProSpec (Rehovot, Israel), and GSH were obtained from Beyotime Institute of Biotechnology (Shanghai, China). The cell images were obtained by a phase-contrast microscope (IMT-2, Olympus, Tokyo, Japan) equipped with a digital microscope camera (PDMC II, Polaroid, Waltham, MA).

### Determination of total sterol contents

Lipid was extracted by the method of Bligh and Dyer [5]. Total sterol contents in the lipid were measured by an enzymatic cholesterol assay kit (Roche Diagnostics, Mannheim, Germany), which determines the levels of 3β-hydroxysterols.

### Analysis of adherent cell number and apoptosis assay

The number of adherent cells after cholesterol loading was analyzed by removing the detached cells by washing the plate once with the medium. The adherent cells were counted after trypsinization and collection by centrifugation.

Apoptosis of MIN6 cells were assessed by the terminal deoxynucleotidyl transferase-mediated deoxyuridine triphosphate-biotin nick end labeling (TUNEL) method using an *in situ *apoptosis kit (Takara, Otsu, Japan). For the detection of FITC fluorescence, the main beam splitter for excitation, the secondary beam splitter for emission, and barrier filter were 488 nm, 570 nm, and 505 nm long pass, respectively. The countstaining was performed using propidium iodide (PI). Procedures for immunocytochemical analysis were described previously [4]. In brief, after fixation and blocking, the cells were incubated with the first antibody against rabbit active caspase-3 (Sigma-Aldrich, St. Louis, Missouri, USA) followed by incubation with anti-rabbit IgG antibody conjugated with Alexa Fluor 488 (Molecular Probes, Eugene, OR). Several images were captured with the same set of optical parameters. The densitometric analysis was performed using a Multi Gauge software in LAS-1000 (Fuji Film).

### Western blot analysis

Procedures for preparation of whole cell lysates and Western blot analysis were described previously [4]. In brief, whole cell lysates (60 μg/lane) were separated by 10% SDS-PAGE, and transferred onto polyvinylidene difluoride membrane (Amersham Pharmacia, Piscataway, NJ). The blots were probed with the first antibodies against p53, followed by incubation with horseradish peroxidase-conjugated anti-rabbit IgG antibody. Rabbit p53 antibody was purchased from Cell Signaling (Beverly, MA). Rabbit anti-actin was from Sigma-Aldrich. The proteins were visualized using enhanced chemiluminescence reagents (Pierce, Rockford, IL). The images of the blotted membranes were obtained by an LAS-1000 lumino-image analyzer (Fuji Film, Tokyo, Japan). Densitometric analysis was performed with the same instrument.

### Measurement of cellular ROS production

Intracellular ROS were measured by a fluorescent dye technique [6]. MIN6 cells were cultured on glass cover-slips and were treated for 30 min with 20 μM 2',7'-dichlorofluorescin diacetate (DCFHDA, Molecular Probes) in phosphate buffered saline (PBS). The cover slips were fixed and mounted. For the detection of fluorescence of 2', 7'-dichlorofluorescein (DCF), the main beam splitter for excitation, the secondary beam splitter for emission, and barrier filter were 488 nm, 570 nm, and 505 nm long pass, respectively. Several images were captured with the same set of optical parameters. The densitometric analysis was performed using a Multi Gauge software in LAS-1000 (Fuji Film).

### Analysis of LDLs and oxidized LDLs by agarose electrophoresis

Oxidation of LDL particles (at 1 mg/ml particles concentration in PBS) was performed by incubation with 5 μmol/l CuSO4 at 37°C for 18 h. The oxidation reaction was stopped for 30 min by adding 300 μmol/l EDTA and by thorough dialysis against PBS and subsequetly against medium without FBS [7]. The electrophoretic mobility of n-LDLs (LDLs in DMEM), LDLs in growth medium and oxidized-LDLs were determined by agarose gel electrophoresis ( 0.5% agarose and 0.1 mol/L barbital buffer containing 4% bovine serum albumin ) under the condition of 200 V, 80 mA in 0.05 mol/L barbital buffer for nearly 0.5 h, then the gel was fixed in methanol for 15 min and stained with oil-red for 16 h After staining, the gel was washed by a bleaching solution (95% ethanol: water = 5:3) [8].

### Statistical analysis

Statistical analysis was performed with ANOVA followed by Bonferroni's multiple t-test, and a p value less than 0.05 was considered statistically significant.

## Results

### LDLs loading induce apoptosis of MIN6s

**Figure 1:**
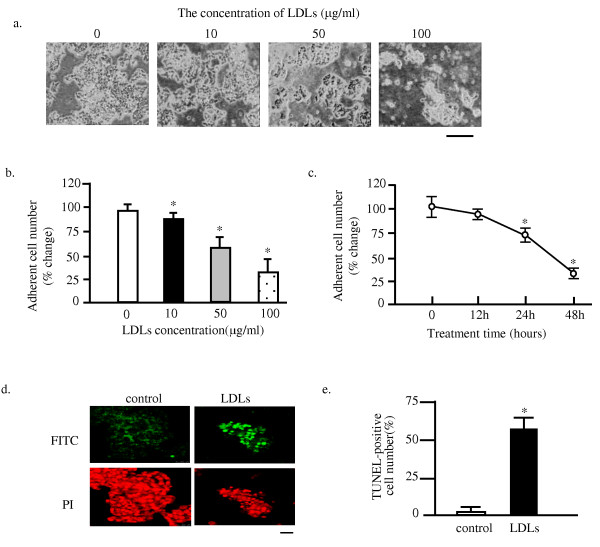
**LDLs induce the apoptosis of MIN6sThe MIN6 cells were exposed to LDLs with indicated concentrations for 48 hours**. The cell images were obtained with a phase-contrast microscope at 48 hours after the exposure. Bar, 25 μm (a). The numbers of adherent cells were expressed as percentage of the number of control cells (b). Mean ± SD (n = 3). *: p < 0.05 vs. the levels of control cells (b and c). DNA fragmentation was analyzed by the TUNEL method at 24 h after LDLs exposure. Representative images are shown. Bar, 25 μm (d). The fold increase in TUNEL positive cells above the control (LDLs-unloaded cells is shown in Figure e. *: p < 0.05.

As shown in Figure 1a, many MIN6 cells were detached within 48 hours after LDLs loading in a dose-dependent manner, a significant decrease being observed from 10 μg/ml of LDLs. On the other hand, most of LDLs-unloaded cells (control) remained adherent. These data demonstrated LDLs loading induces deleterious effect on cell adherence of MIN6 cells in dose-dependent manners. Statistical analysis demonstrated that decrease in the adherent cell number induced by LDLs loading was significant (Figure 1b). The cytotoxity of LDLs to MIN6s also revealed the time-dependent manner, demonstrated the statistical result, obtained from the cell cultured with 100 μg/ml of LDLs (Figure 1c).

To determine whether the cell death of MIN6s was because of apoptosis, both LDLs -loaded and non-loaded cells cultured on the coverslips were subjected to the TUNEL staining (Figure 1d). Only few TUNEL-positive cells were detected in control cells, whereas about 60% of the adherent cholesterol-loaded cells were positively stained at 48 h. This increase in the number of TUNEL-positive cells was significant after LDLs-loading, if compared to that of control (Figure 1e).

**Figure 2:**
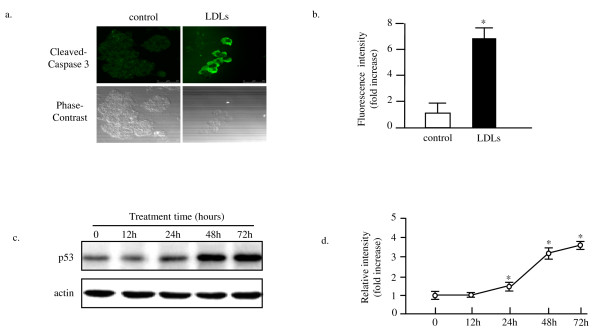
**LDLs exposure activates the apoptotic signaling pathway**. At 48 hours after exposure to 100 μg/ml of LDLs, the cells cultured on the coverslips were fixed and subjected to immunocytochemistry with the first antibody against rabbit active caspase-3 and the second rabbit IgG conjugated with Alexa fluor 568. Representative images are shown. Bar, 25 μm (Figure 2 a ). Whole cell lysates were prepared at 0, 12, 24, 48 and 72 hours after the exposure to the 100 μg/ml of LDLs, and subjected to Western blot analysis using antibodies against p53 and actin. The representative images are shown in Figure 2c. The quantitative results are shown in Figure 3 b and d. The p53 levels were normalized by the actin levels in Western blotting. The changes under the LDLs exposure are expressed as relative changes of the level at 0 time point. Mean ± SD (n = 3). *, #: p < 0.05 vs. the 0 point.

We next investigated the molecular mechanism of the LDLs-induced apoptosis of β cells. The immunocytochemical analysis using the antibody against the cleaved-caspase 3 revealed that the fluorescent intensity in the LDLs exposure group was much stronger than that of control, suggesting that the apoptosis of MIN6s induced by LDLs was caspase 3-dependent (Figure 2 a, b). The Western blot analysis utilizing the antibody against p53 showed that the p53 was upregulated significantly after the LDLs exposure 24 and 48 hours (Figure 2 c, d). This result suggested that the p53 was involved in the LDLs-induced apoptosis of MIN6 pancreatic cells.

### Oxidative stress contributes to cholesterol-induced apoptosis of MIN6s

**Figure 3:**
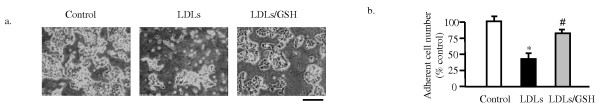
**GSH reverses the apoptosis induced by LDLs in MIN6s**. The MIN6 cells were exposed to the 100 μg/ml of LDLs alone or the combination of LDLs and 2 mM of reduced glutathione (LDLs/GSH) for 48 hours. The cell images were obtained with a phase-contrast microscope at 48 hours after the exposure. Bar, 25 μm (a). The numbers of adherent cells were expressed as percentage of the number of control cells (b). Mean ± SD (n = 3). *: p < 0.05 vs. the levels of LDLs loaded-cells.

Several reports have indicated a link between high dietary fat exposures to oxidative stress in animal model. These studies showed an up-regulation of ROS production, lipid peroxidation, and oxidized nucleotides and proteins, reflecting the increased oxidative stress in response to high fat diet [9,10]. To study the possible effect of LDLs accumulation on ROS generation in MIN6 cells, the effect of reduced form of glutathione (GSH) on protection of β cells from LDLs-induced apoptosis was investigated. As shown in Figure 2a and 2b, the adherent cell number of LDLs/GSH group was significantly increased compared to that of LDLs-loaded cells. This suggested that the excess ROS generation after LDLs loading might contribute to the apoptosis. To confirm further this possibility, the intracellular ROS levels were measured after exposure to LDLs, utilizing a cell permeable indicator for ROS, DCFHDA. As shown in Figure 3a and 3b, confocal microscopic images revealed that LDLs loading markedly increased the fluorescent intensity in most of MIN6 cells while much weaker signal was observed in control. This effect of LDLs loading persisted for 12 hours. The generation of excess ROS due to overproduction will damage DNA, protein, and membrane lipids [11]. In addition to such direct effects on cell components, ROS excess has been demonstrated to affect the intracellular signaling cascades that involve stress activated protein kinases (SAPKs), resulting in apoptosis [12].

### Accumulation of cellular cholesterol might involve in the LDLs-induced apoptosis

**Figure 4:**
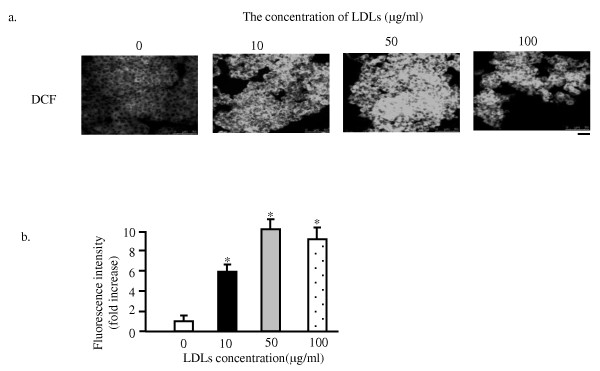
**LDLs loading induces the excess generation of ROS**. The MIN6 cells were exposed to the LDLs with indicated concentrations for 48 hours. Then the cells were subjected to the incubation with 20 μM DCFHDA, for 30 minutes at 37°C, followed by fixation and mounting. The cell images were obtained with confocal laser microscope. Bar, 25 μm (a). Fluorescent intensities of DCF (2',7 ' -dichlorofluorescein) are expressed as fold increases of the of control level (b). Mean ± SD (n = 20). *: p < 0.05 vs. the levels of control. #: p < 0.05 vs. the levels of LDLs-loaded cells.

Our previous study has demonstrated that free cholesterol could induce apoptosis of MIN6s through activation of oxidative stress pathway [13]. In order to test whether LDLs toxicity in MIN6s affected the intracellular cholesterol level, we measured the concentration of cholesterol of control and LDLs-loaded cells by the enzymatic cholesterol assay kit. The results are as shown in Figure 4, when the MIN6 cells were exposed to 50 μg/ml of LDLs for 48 hours, the extracellular cholesterol in growth medium was about 160 μM, that was significant higher than that of control (about 100 μM (Figure 4a). The LDLs treatment also significantly increased the intracellular cholesterol level up to 10.5 μmol/4 × 10^7 ^cells, if compared to the 8 μmol/4 × 10^7 ^cells in control (Figure 4b). These data were similar to those obtained from the platelets prepared from healthy control and diabetic patients (11.9 and 15.6 μmol/4 × 10^7 ^cells, respectively) [14]. Our previous study using the water-soluble cholesterol also showed the intracellular cholesterol level was increased up to 9.6 μmol/4 × 10^7 ^cells by free cholesterol loading in MIN6s [14]. This result suggested that the LDLs-loading-induced apoptosis of MIN6s might be cause by the accumulation of intracellular cholesterol that activates the stress signaling and result in apoptosis.

### The native-LDLs, but not oxidative-LDLs induces the apoptosis of MIN6s

**Figure 5:**
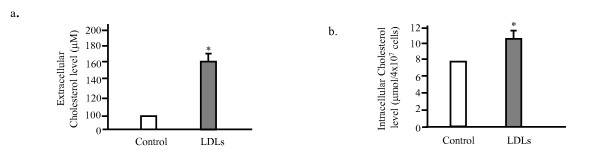
**The LDLs loading induces accumulation of intracellular cholesterol**. MIN6 cells were exposed to CLC with 250 times dilution or 80 μM of LDLs in the growth medium. The medium was subjected directly to the extracellular cholesterol measure (a). The cells then were harvested and counted. The total lipids were extracted from cells and subjected to the intracellular cholesterol measurement (b). Mean ± SD (n = 3). *: p < 0.05 vs. the levels of control.

There are some of studies showing that the oxidized LDLs could result in the death of pancreatic β cells. Here we observed the native-LDLs without oxidative modification induced the apoptosis of MIN6s. To confirm whether the LDLs added in the growth medium after 48 hours incubation is oxidized in the present study, we performed the agarose electrophoretic analysis. As shown in Figure 5, *in vitro *oxidized LDLs by CuSO4 (Line 3) decreased the electrophoretic mobility of LDLs, compared to the native LDLs control (Line 1). The electrophoretic mobility of LDLs directly added in growth medium for 48 hours (Line 2) was similar to that of the stock LDLs. This result demonstrated that the LDLs added into the medium was not oxidized after long time exposure to MIN6.

## Discussion

**Figure 6:**
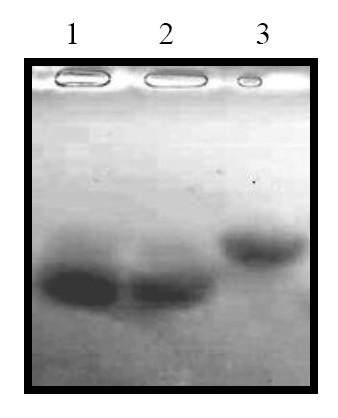
**Long time exposure to MIN6 cells does not oxidize LDLs**. MIN6 cells were exposed to the 100 μg/ml of LDLs for 48 hours in the growth medium, and then the growth medium containing LDLs was collected and concentrated (line 2). On the other hand, the LDLs were also prepared by direct dilution with DMEM from stock solution (line 1). The oxidized LDLs were prepared as described in Materials and Methods (line 3). All three types of LDLs (about 1mg/ml protein) were subjected to agarose gel electrophoresis followed by the gel staining with oil-red.

The present study for the first time demonstrated that the intracellular cholesterol accumulation induced by LDL particles loading induces apoptosis of MIN6 pancreatic β cells (Figure 1 and 5). The LDLs-induced apoptosis was through the activation of caspase 3 and associated with upregulation of p53 (Figure 2). This apoptosis might be because of the generation of excess of reactive oxygen species, suggested by that the reduced form of glutathione (GSH) could rescue the MIN6s from apoptosis induced by LDLs (Figure 3). This is again confirmed by determining of the intracellular ROS level utilizing the fluorescent dye DCFHDA. It was demonstrated that the LDLs loading induced the generation of excess of intracellular ROS (Figure 4). We next investigated whether LDLs is modified by oxidation after addition into the growth medium for long time. The results clearly demonstrated that the LDLs loaded into medium of MIN6s for 48 hours were not oxidized (Figure 6).

Our data have demonstrated for the first time that the LDLs induce beta cell apoptosis because of the excess cholesterol other than its oxidative modification. This is similar to the previous study. Similar results were reported by Miriam Cnop *et al.*, demonstrating that the LDL particles (from 6 μg/ml on), but not oxidized LDLs, caused the necrosis of primary cultured β-cells within 2 days. However, they could not detect the apoptosis under those conditions [2]. The apoptosis of MIN6 pancreatic cells after loading LDLs on the similar range was observed in the present study, demonstrated by sensitive TUNEL *in situ *apoptotic assay. The difference might because of the sensitivity of apoptosis detection apoptotic detective methods.

Our results contradict some of studies showing that non-oxidized LDLs do not induce β cell apoptosis. We think this is might be cause by different system used. Most of studies showing that non-oxidized LDLs do not induce β cell death were performed using the primary culture of mice or human islets. For successful primary culture, they have to culture the islets on extracellular matrix-coated plates. It is reported that the extracellular matrix could inhibit apoptosis by a NF-κB-dependent mechanism [14]. Our previous experience of neuronal primary culture also suggests it. It is likely that it is difficult to detect the apoptotic cells when the cells were cultured on the extracellular matrix-coated plates. We obtained the data from β cell line-MIN6, cultured on the plate without any coating. On the other hand, the LDLs loaded into growth medium containing 10% FBS should be protected from the air-oxidation, because there are many components in serum. This is demonstrated by our results using gel electrophoretic analysis (Figure 5). We observed that LDLs directly induced the beta cell apoptosis without any modification. Taken together, we believe that non-oxidized LDLs could induce beta cell apoptosis.

How LDL particles loading induce oxidative stress is still unclear. Our data suggested that the LDLs loading would generate the accumulation of intracellular cholesterol, which might induce the oxidative stress, demonstrated by our previous study. Emerging evidence indicates that cholesterol-rich microdomains of plasma membrane, lipid rafts and/or caveolae may be critically involved in distal redox-sensitive signaling events and ultimate cell fate. Zhang et al elegantly demonstrated that death receptor ligands and apoptotic factors stimulate lipid raft clustering, which results in aggregation and activation of NAD (P) H oxidase and consequent endothelial dysfunction [15]. Hence, lipid rafts are implicated in both growth and death signaling through NAD (P) H oxidase-generated ROS. It has been reported that cholesterol accumulation in the plasma membrane promotes formation of membrane lipid raft [16]. Taken together, it is suggested that the apoptosis of MIN6 cells induced by LDLs loading might be mediated through promoted formation of lipid raft, resulting in the activation of NAD (P) H oxidase and generation of ROS.

In conclusion, the present study demonstrated for the first time that LDL particle loading results in accumulation of intracellular cholesterol and induces apoptosis of MIN6s through generation of ROS. Our finding provides direct evidence at the cellular level that hypercholesterolemia under diabetic conditions impairs not only cardiovascular but also pancreatic β cell functions. This indicates that prevention of hypercholesterolemia could be useful in treating T2D.

## List of abbreviations

T2D: Type 2 Diabetes; LDL: low density lipoprotein; LDLs: Low density lipoprotein particles; ROS: Reactive Oxygen Species; GSH: Glutathione; MIN6s: MIN6 pancreatic β cells; PI: propidium iodide; DCFHDA: 2',7'-dichlorofluorescin diacetate; SAPKs: stress activated protein kinases.

## Competing interests

The authors declare that they have no competing interests.

## Authors' contributions

XL and BG conceived the idea and designed the study. JL and XC performed the analysis of LDLs cytotoxicity in MIN6s by phase-contrast microscopy and TUNEL in situ apoptotic assay.  XH and  XW performed the measure of intracellular ROS level using the DCFHDA fluorescent probe. CZ and YW carried out the analysis of oxidative modification of LDLs by agarose electrophoresis. YL performed the oil red staining. SH provided critical corrections and language polishing to the manuscript. All authors read and approved the final manuscript.
